# Commentary: A Supraventricular Tachycardia: What Is It? Where Should One Ablate?

**DOI:** 10.19102/icrm.2017.080403

**Published:** 2017-04-15

Here, several experts discuss the case of supraventricular tachycardia presented by Drs. Chaudhari and Olshansky.

## Dr. Katritsis explains


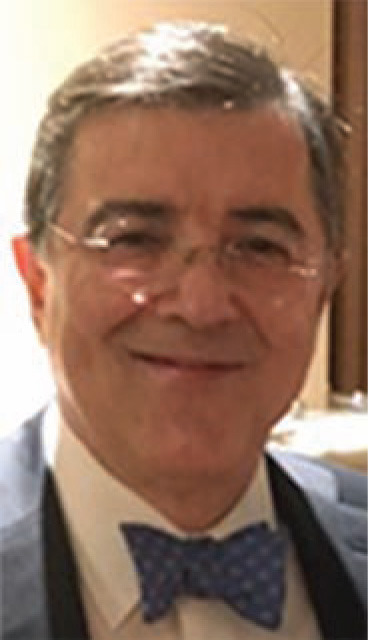


**Figure 2:**
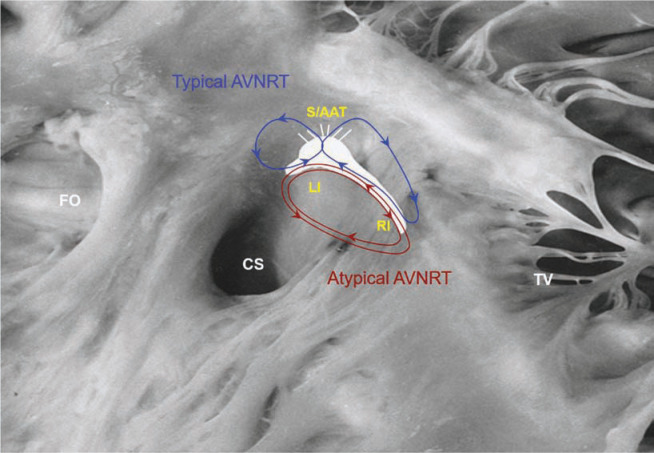
The occurrence of AV block during AVNRT without any recording of the activation of the His-bundle can be explained by proximal intra-Hisian block.

The prolonged AH that allows for 1:1 conduction is presumably due to a shift in the activation of the left nodal extension (as a slow pathway) to the right (as a slow pathway), which is longer. The mechanism for resumption of 1:1 is either due to the use of the right-sided atrial input, which is longer than the left **(Figure 2)**, or it could be due to prolongation of conduction in the same atrial extension (slow pathway) that allows for 1:1 conduction down the His-bundle. Furthermore, atrioventricular (AV) block during atrioventricular nodal re-entrant tachycardia (AVNRT) without any visible recording of the activation of the His-bundle can also be explained by proximal Intra-Hisian block.

Demosthenes Katritsis, MD, PhD (London), FRCP, FESC, FACC

E-mail: dkatrits@dgkatritsis.gr

www.dgkatritsis.gr

Department of Cardiology

Athens Euroclinic

Athens, Greece

## Figures and Tables

**Figure 3:**
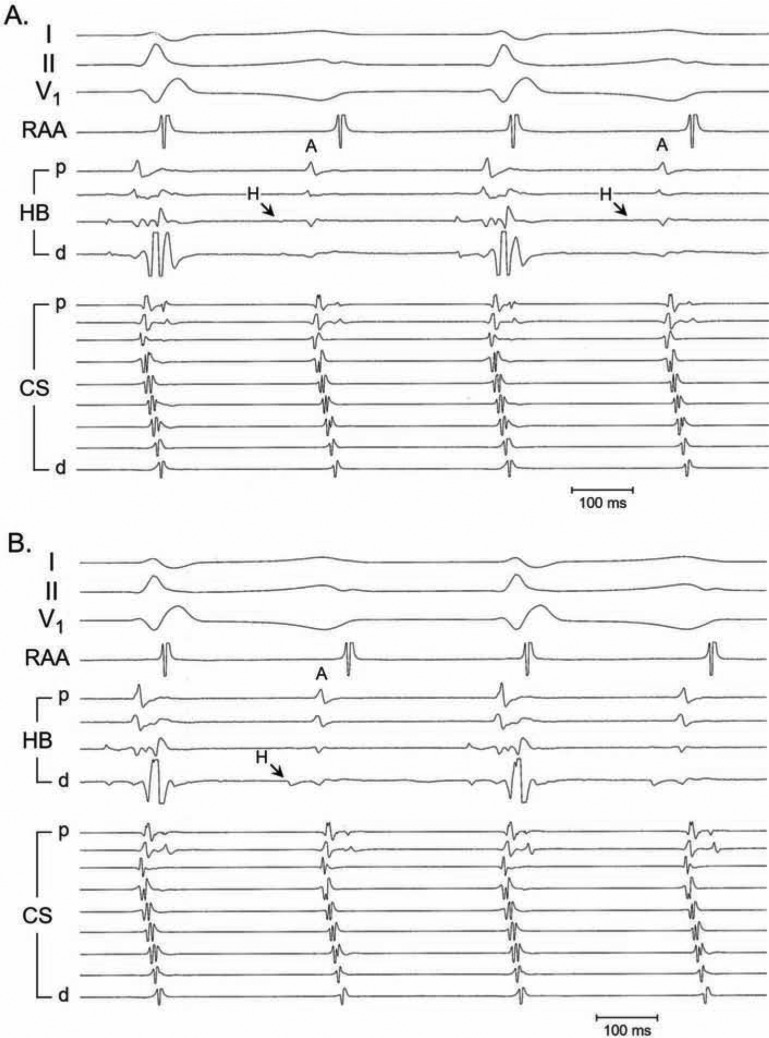
Slow/fast AVNRT with 2:1 block distal to the most proximal His-bundle potential, recorded in a 19-year-old woman with a 1-year history of episodes of supraventricular tachycardia. (a) During the blocked complexes, the two most distal His-bundle electrograms record a tiny, far-field His-bundle potential. This potential may be easily overlooked to suggest that block occurred proximal to the H potential. (b) Repositioning the His-bundle catheter allowed for clear recording of the proximal H potential during the blocked complexes.

## Drs. Verma and Knight comment

The original report of 2:1 AV block during AVNRT was published over 40 years ago.^1^ This phenomenon has been demonstrated in approximately 9% of adult cases, and up to 17% of pediatric cases.^2,3^ The likelihood of this occurring in the electrophysiology laboratory appears higher than spontaneously, perhaps because of the artificial means of induction.

There has been a debate with regards to the mechanism and location of block in these cases. In up to 60% of cases of 2:1 AVNRT, a His-bundle potential can be recorded on non-conducted beats. This led to the argument that in cases with no His-bundle potential, the mechanism was AV nodal block, while in cases in which a His-bundle potential is seen, the mechanism was intra- or infra-His-bundle block.

The lack of a proximal His-bundle recording shown on the non-conducted beats does not rule intra-Hisian block to be proximal to the recording. Resolution with a pacing stimulus (or premature ventricular contraction (PVC) in this case) strongly supports function block in the HP system, rather than rate-related 2:1 block in the distal AV node region.

However, the elegant study by Man et al. convincingly demonstrated that the location of block in all cases was at or below the His-bundle.^2^ The mechanism appears to be functional block due to a “long-short” sequence into the His-bundle. Atropine, which improves AV nodal conduction without affecting His-bundle conduction, never resulted in the restoration of 1:1 conduction, arguing against AV nodal block as the mechanism. However, an appropriately timed single ventricular extrastimulus resolved the 2:1 conduction in all cases, including those without a discernable His-bundle recording. This results from retrograde depolarization of the His-bundle (or below) to resolve the functional block and allow for 1:1 conduction. Resolution of 2:1 conduction in this manner would not be expected to occur in the setting of AV nodal block. This is especially true in the setting of a His-bundle refractory PVC, as in the above example, as there is no AV nodal depolarization. Though 2:1 AVNRT is uncommon, it is an important entity to recognize, since failure to do so could result in errors in mapping and ablation, resulting in significant harm.

Nishant Verma, MD

E-mail: nverma1@nm.org

Division of Cardiology Department of Medicine

Northwestern University Feinberg School of Medicine

Chicago, IL 60611

Bradley Knight, MD

E-mail: Bradley.Knight@nm.org

Division of Cardiology Department of Medicine

Northwestern University Feinberg School of Medicine

Chicago, IL 60611

## Dr. Jackman comments

I agree completely with the explanation from Drs. Verma and Knight. During slow/fast AVNRT with 2:1 AV block, one can essentially always find a tiny, very proximal, His-bundle potential, indicating block distal to the AV node **(Figure 3)**. However, it often requires careful positioning of the His-bundle catheter to find the tiny, rounded proximal “H” potential. In **Figure 1** *(found in “A Supraventricular Tachycardia: What Is It? Where Should One Ablate?”),* the His-bundle catheter is probably positioned too distally (sharp ventricular potential and far-field atrial potential on the proximal His-bundle electrogram), and is likely recording the proximal right bundle branch. The sharp part of the normal “His-potential” is generated by the proximal right bundle branch, usually recorded at around 12 ms after the onset of the tiny, rounded His-bundle potential.

Warren Jackman, MD

E-mail: warren-jackman@ouhsc.edu

Heart Rhythm Institute

University of Oklahoma College of Medicine

Oklahoma City, OK 73104

## Dr. Tchou discusses

I am not sure that the PVC has anything to do with the recovery of conduction. Typically, when 2:1 block occurs during AVNRT, it is a block that occurs within the His-bundle, though the exact site may vary. This is certainly what is happening here with the 2:1 block. When the 2:1 block is resolved with a PVC, the PVC must pre-excite the His-bundle just distal to the site of 2:1 block. This would shorten the His-bundle refractoriness distal to the site of the block and allow for the next His-bundle activation to conduct.

The mechanism of the 2:1 block has to do with the long cycle length below the block site, which keeps the His-Purkinje action potential there long enough such that the short cycle length of the blocked beat coming down meets refractory His-Purkinje tissue distal to the site of the block. What happens when a PVC occurs is that it propagates retrogradely into the His-Purkinje system (HPS) (if it is not too premature). This propagation shortens the cycle length of the HPS just distal to the site of the block, thus shortening the action potential duration (and refractoriness) of the HPS distal to the site of the block. This shortening then allows for the next beat that would have blocked to instead conduct and resolve the block. In the example shown in **Figure 1** *(found in “A Supraventricular Tachycardia: What Is It? Where Should One Ablate?”)*, the mechanism of resolution is likely due to slowing of the cycle length of tachycardia, as well as a component of spontaneous resolution of 2:1 block that frequently occurs. Several observations suggest that PVC has nothing to do with resolving the HPS system refractory period. These are:

1. There is spontaneous slowing of the atrial cycle length (CL) that can be seen beginning with the fourth atrial beat on.
2. The PVC does not conduct retrogradely, even to the distal portions of the His-bundle recording site, so it is unlikely to have influenced the HPS near the site of the block, which is high, above the site of His-bundle recording. The His-bundle recording is more distal, as there is virtually no A on the electograms. During the blocked atrial beat, there is no His-bundle recording at all. So, the 2:1 block site is above the recording site of the His. The PVC does not influence the antegrade conduction of the His (seen after the Vof the PVC). In fact, that His-bundle recording occurs just in time. The HH interval is equal to the corresponding AA intervals (in that the second and third visible H interval is the same as the corresponding AA interval, even though the CL slows a little). Thus, the blocked His-bundle (the third visible His-bundle) is blocked below the recording site of the H because of interference from the PVC that blocked distally in the HPS (i.e. persistent refractoriness in the distal HPS because of retrograde penetration of the PVC into the distal HPS.)
3. The fourth H should have been the blocked beat (per the 2:1 pattern), but it conducted past the site of the block (which is up high, above the site of His-bundle recording.) Since the PVC blocked retrogradely very distally in the HPS below the His-bundle recording site, it likely did not influence the site of antegrade block, which was quite high above the His-bundle recording site.
4. The likely cause of the resolution of 2:1 block is 1) these occur spontaneously all the time; and 2) there is a slowing of the AA cycle length that may allow for the resolution to occur when the beat is now outside of the HPS refractoriness.

Everything considered, this is a very interesting tracing. At first glance, I thought that it was a standard resolution of 2:1 conduction during AVNRT brought on by a PVC. But on further examination, the timing of the PVC and its retrograde conduction characteristics would likely not have allowed it to influence the resolution of the block. So, I would call this spontaneous resolution of the 2:1 in part facilitated by slowing of the tachycardia. The PVC is a bystander in this case.

I agree with Dr. Katritis. I am not sure that I am convinced that there is a visible atrial activation on the His-bundle recording, but perhaps the ablation recording is at the mid-septum. Shifting from one slow pathway to another is certainly a possibility when there is a changing AH interval. Prolongation of conduction down the same path is also possible.

I agree with Dr. Jackman that 2:1 block in AVNRT is always due to Hisian block. The physiology of nodal conduction tissue would make it unlikely that it can generate 2:1 functional block that would resolve with a premature depolarization. This is quite feasible with the electrophysiology of the HPS.

I agree with Drs. Verma and Knight, except when they suggest that the “His-bundle refractory PVC” is the proximal cause of the resolution of 2:1 block. This PVC blocks very distally in the HPS. So, it is unlikely to affect the His-bundle refractoriness by premature activation at the site of antegrade block, which is well above the His-bundle recording site (in that not even a far-field His-bundle is seen). Even if one were to propose an electronic effect, the site of retrograde block of the PVC in the HPS and the site of antegrade block during tachycardia are separated enough to make that unlikely.

A His-bundle refractory PVC typically has to propagate up the HPS system and collide with the oncoming His-bundle activation of a conducted beat near the site of the block. In that manner, the retrograde activation pre-excites the portion of HPS distal to the site of block. This pre-excitation result is the peeling back of the refractoriness distal to the site of the block because of earlier activation and because of the cycle length-related shortening of the action potential duration.

Thus, the following beat would find the HPS distal to the site of block no longer refractory. In this case, if there is a collision at all of the PVC with the antegrade His-bundle, it is very distal and not anywhere near the site of block. I think spontaneous resolution of the 2:1 block, in part facilitated by a longer AH interval, is the cause of block resolution in this example.

Patrick Tchou, MD

E-mail: tchoup@ccf.org

Section of Cardiac Electrophysiology and Pacing

Department of Cardiovascular Medicine

Heart and Vascular Institute

Cleveland Clinic

Cleveland, OH 44195

## Dr. Efimov provides insights

I agree with what has been said. However, this case highlights an idea that we have presented based on both molecular and functional data. Hucker et al.^1^ presented three-dimensional reconstruction of the human AV junction labeled with Cx43 antibodies. This study presented evidence of strikingly differential expression of Cx43 between the slow and fast pathways, which persisted along the AV conduction axis, including the compact AV node and the bundle of His. A subsequent study from our group demonstrated that His-bundle electro-gram amplitude and morphology differed greatly depending on whether the slow or fast pathway served as the entry point to the longitudinally dissociated AV node, and corresponding domains of the His-bundle.^2^ This study suggested that not all His-bundle responses are equal: the bundle of His comprises at least two distinct domains (Cx43-positive and Cx43-negative) developmentally and electrically coupled to the slow and fast pathways, respectively. The difference in His-bundle electrogram amplitude produced by the two domains could differ by an order of magnitude. High resolution electrophysiology mapping is needed to define the two domains of the His-bundle, as the two different domains could have different conduction properties and/or refractory periods; additionally, perhaps only one of them is in fact safely coupled to the ventricles. Moreover, quantitative three-dimensional immunohistochemistry of the human AV junction, in combination with CLARITY methodology, will help to dissect the human AV junction as well as its structure and protein expression.

Igor Efimov, PhD

E-mail: efimov@gwu.edu

Department of Biomedical Engineering

George Washington University

Washington, DC 20052

## Closing comments

A superb discussion from the Masters!

2:1 AVNRT can mimic atrial tachycardia or atrial flutter, but it is important to recognize it and treat it with ablation lesions directed at the correct location. Beware, as it may be important in your next ablation. We hope the comments from the experts provide insight into the phenomena seen here. We welcome your comments, and look forward to your upcoming Unicorn contributions.

Brian Olshansky, MD, FHRS, FACC, FAHA

Professor Emeritus

University of Iowa

Cardiac Electrophysiologist

Mercy Hospital-North Iowa

Mason City, Iowa 50401

## References

[1] Wellens HJ (1975). Unusual examples of supraventricular re-entrant tachycardias. Circulation..

[2] Man KC, Brinkman K, Bogun F, Knight B, Bahu M, Weiss R (1996). 2:1 atrioventricular block during atrioventricular node reentrant tachycardia. J Am Coll Cardiol..

[3] Mahajan T, Berul CI, Cecchin F, Triedman JK, Alexander ME, Walsh EP (2008). Atrioventricular nodal reentrant tachycardia with 2:1 block in pediatric patients. Heart Rhythm..

